# Blood–brain barrier P-glycoprotein function in healthy subjects and Alzheimer's disease patients: effect of polymorphisms in the ABCB1 gene

**DOI:** 10.1186/2191-219X-2-57

**Published:** 2012-10-16

**Authors:** Daniëlle ME van Assema, Mark Lubberink, Patrizia Rizzu, John C van Swieten, Robert C Schuit, Jonas Eriksson, Philip Scheltens, Matthias Koepp, Adriaan A Lammertsma, Bart NM van Berckel

**Affiliations:** 1 Department of Neurology and Alzheimer Center, VU University Medical Center, P.O. Box 7057, Amsterdam, 1007 MB, The Netherlands; 2 PET Centre, Uppsala University Hospital, Uppsala, S 751 85, Sweden; 3 Department of Clinical Genetics, VU University Medical Center, P.O. Box 7057, Amsterdam, 1007 MB, The Netherlands; 4 Department of Neurology, University Medical Center Rotterdam, P.O. Box 2040, Rotterdam, 3000 CA, The Netherlands; 5 Department of Nuclear Medicine and PET Research, VU University Medical Center, P.O. Box 7057, Amsterdam, 1007 MB, The Netherlands; 6 Institute of Neurology, University College London, London, WC1N 3BG, UK

**Keywords:** Blood–brain barrier, P-glycoprotein, ABCB1, MDR1, Polymorphisms, (*R*)-[^11^C]verapamil, PET

## Abstract

**Background:**

P-glycoprotein is a blood–brain barrier efflux transporter involved in the clearance of amyloid-beta from the brain and, as such, might be involved in the pathogenesis of Alzheimer's disease. P-glycoprotein is encoded by the highly polymorphic ABCB1 gene. Single-nucleotide polymorphisms in the ABCB1 gene have been associated with altered P-glycoprotein expression and function. P-glycoprotein function at the blood–brain barrier can be quantified *in vivo* using the P-glycoprotein substrate tracer (*R*)-[^11^C]verapamil and positron emission tomography (PET). The purpose of this study was to assess the effects of C1236T, G2677T/A and C3435T single-nucleotide polymorphisms in ABCB1 on blood–brain barrier P-glycoprotein function in healthy subjects and patients with Alzheimer's disease.

**Methods:**

Thirty-two healthy subjects and seventeen patients with Alzheimer's disease underwent 60-min dynamic (*R*)-[^11^C]verapamil PET scans. The binding potential of (*R*)-[^11^C]verapamil was assessed using a previously validated constrained two-tissue plasma input compartment model and used as outcome measure. DNA was isolated from frozen blood samples and C1236T, G2677T/A and C3435T single-nucleotide polymorphisms were amplified by polymerase chain reaction.

**Results:**

In healthy controls, binding potential did not differ between subjects without and with one or more T present in C1236T, G2677T and C3435T. In contrast, patients with Alzheimer's disease with one or more T in C1236T, G2677T and C3435T had significantly higher binding potential values than patients without a T. In addition, there was a relationship between binding potential and T dose in C1236T and G2677T.

**Conclusions:**

In Alzheimer's disease patients, C1236T, G2677T/A and C3435T single-nucleotide polymorphisms may be related to changes in P-glycoprotein function at the blood–brain barrier. As such, genetic variations in ABCB1 might contribute to the progression of amyloid-beta deposition in the brain.

## Background

P-glycoprotein (Pgp) is a 170 kDa membrane-bound efflux transporter located in several organs throughout the body with an excretory and/or barrier function, such as the liver, kidneys, intestine, placenta, testes and the blood–brain barrier (BBB) [1]. At the BBB, Pgp is highly expressed and has an important function in the transport of a wide variety of endogenous and exogenous substances out of the brain into the bloodstream [2]. Pgp has been shown to play a role in amyloid-beta clearance from the brain and, as such, has been hypothesized to be involved in the pathogenesis of Alzheimer's disease (AD) [3]. Recently, BBB Pgp dysfunction in AD patients was shown using the Pgp substrate tracer (*R*)-^11^C]verapamil and positron emission tomography (PET) [4].

Pgp is encoded in the ABCB1 gene (formerly known as the multidrug resistance (MDR1) gene), which spans 28 exons that code for 1280 amino acids and is located on chromosome 7q21 [5]. The ABCB1 gene is highly polymorphic, and to date, over a hundred single-nucleotide polymorphisms (SNPs) of this gene have been discovered [5]. The majority of SNPs are either intronic or non-coding. Only a minority of variants in the coding region lead to a change in amino acids [5,6].

In Caucasian subjects, high SNP frequencies in ABCB1 were observed for 3435T in exon 26 (54%) and 2677G in exon 21 (56%). High SNP frequencies were also found for 2677T (42%) and 1236T in exon 12 (46%) [5,7]. Of the 15 most frequently occuring SNPs, the synonymous variant (no amino acid exchange) C1236T (rs1128503), the non-synonymous variant G2677T/A (amino acid exchanges Ala893Ser or Ala893Thr) (rs2032582) and the synonymous variant C3435T (rs1045642) are most widely studied regarding their consequences for Pgp function and/or expression [5,6].

Altered transport function of Pgp was observed for the C3435T SNP, as the 3435T allele was associated with decreased Pgp expression in the duodenum, and increased oral bioavailability of the Pgp substrate digoxin [8]. On the other hand, others could not replicate these findings, and even opposite effects of this SNP have been shown [9,10]. Whilst *in vitro* studies did not find effects of the G2677T/A SNP [11,12], another study showed an association with decreased Pgp expression in the human placenta [13].

Highly significant linkage disequilibrium was shown among exons 12 C1236T, 21 G2677T and 26 C3435T, which could account for most of the haplotypes (combinations of SNPs) seen in ABCB1 [13-15]. To date, two studies have assessed the effects of ABCB1 haplotypes on BBB Pgp function using PET and ^11^C]verapamil. In these studies, healthy subjects with the homozygous TTT haplotype (1236T, 2677T and 3435T) did not differ in brain distribution of ^11^C]verapamil from subjects with the homozygous CGC haplotype (1236C, 2677G and 3435C) [16,17].

As Pgp is involved in amyloid-beta clearance at the BBB, genetic variations in ABCB1 might be related to an inherited change in the risk of developing AD [3]. Analysis of ABCB1 SNPs in combination with *in vivo* measurement of BBB Pgp function in AD is needed to further evaluate this hypothesis. Therefore, the purpose of this study was to assess the effects of C1236T, G2677T/A and C3435T SNPs on BBB Pgp function in both AD patients and healthy subjects.

## Methods

### Participants

Thirty-two healthy controls and seventeen AD patients were included in this study. All participants underwent a standardized clinical assessment, including medical history, family history, use of medication and drugs of abuse and physical and neurological examinations. All subjects had normal scores on screening laboratory tests. Medication at the time of scanning was not allowed, except for medication known not to interfere with Pgp function [18,19]. Controls fulfilled research criteria of never having been mentally ill, and they had normal brain magnetic resonance imaging (MRI) scans and Mini-Mental State Examination (MMSE) scores within the normal range (MMSE > 26) [20]. For AD patients, a clinical diagnosis of probable AD was established by consensus in a multidisciplinary meeting according to the criteria proposed by the National Institute of Neurological and Communicative Disorders and Stroke and the Alzheimer's Disease and Related Disorders Association [21]. Increased cortical accumulation of ^11^C]PIB PET was required to confirm presence of amyloid pathology in the brain [22]. Written informed consent was obtained from all participants after a complete written and verbal description of the study, and the study was approved by the Medical Ethics Review Committee of the VU University Medical Center.

### MRI

Participants underwent structural MRI scans using either a 1.0-T Magnetom Impact scanner (Siemens Medical Solutions, Erlangen, Germany) or a 1.5-T Sonata scanner (Siemens Medical Solutions). The scan protocol included an identical coronal T1-weighted 3-D magnetization-prepared rapid acquisition gradient-echo sequence, which was used for co-registration and region-of-interest (ROI) definition.

### PET

All PET scans were acquired using an ECAT EXACT HR+ scanner (Siemens/CTI, Knoxville, TN, USA) [23]. Participants received an indwelling radial artery canula for arterial sampling and a venous canula for tracer injection. After a 10-min transmission scan in 2D acquisition mode, which was used to correct the subsequent emission scan for photon attenuation, a dynamic 3D emission scan was started simultaneously with the injection of 369 ± 19 MBq (*R*)-^11^C]verapamil, administered using an infusion pump (Med-Rad, Beek, The Netherlands). The 60-min emission scan consisted of 20 frames with progressive increase in frame duration. Arterial blood was withdrawn continuously using an online sampling device (Veenstra Instruments, Joure, The Netherlands) [24]. At set times, continuous sampling was interrupted briefly, and manual samples were taken. A detailed description of scanning and sampling procedures has been reported previously [4].

### PET data analysis

All PET sinograms were corrected for dead time, tissue attenuation, decay, scatter and randoms. PET scans were reconstructed using a standard filtered back projection algorithm, applying a Hanning filter with a cut-off at 0.5 times the Nyquist frequency. A zoom factor of 2 and a matrix size of 256 × 256 × 63 were used, resulting in a voxel size of 1.2 × 1.2 × 2.4 mm and a spatial resolution of approximately 6.5 mm full-width at half-maximum at the centre of the field of view. Co-registration of structural T1 MR images with corresponding PET images and segmentation of the co-registered MRI into the grey matter, white matter and cerebrospinal fluid was performed using statistical parametrical mapping (SPM, version SPM2, http://www.fil.ion.ucl.ac.uk/spm, Institute of Neurology, London, UK). ROIs were defined on the basis of the segmented MRI and a probabilistic template as implemented in PVElab [25]. ROIs were mapped onto dynamic PET images, generating regional time-activity curves. A global cortical region was defined consisting of the volume-weighted average of the frontal, parietal, temporal and occipital cortices and posterior and anterior cingulate regions [4].

The original on-line blood curve was calibrated using whole blood radioactivity concentrations derived from the manual samples. The calibrated whole blood curve was multiplied with a single-exponential fit to the plasma-to-whole blood ratios of these samples, thereby generating a total plasma curve. Finally, the metabolite-corrected plasma input function was obtained by multiplying this total plasma curve with a sigmoid fit to one minus the polar metabolite fraction [26,27].

Kinetic analyses were performed using software developed within Matlab 7.04 (The Mathworks, Natick, MA, USA). (*R*)-^11^C]verapamil data were analysed using a previously validated constrained two-tissue compartment plasma input model [28]. The non-displaceable binding potential (BP_ND_) was used as outcome measure. Again, data analysis and kinetic modeling procedures have been described in detail elsewhere [4].

### DNA isolation and molecular analysis

The DNAs of 49 samples were isolated from 4 mL of frozen blood samples using the Gentra Puragene Blood Kit (Qiagen, Hilden, Germany) according to the manufacturer's protocol. C1236T, G2677T/A and C3435T polymorphisms were amplified by polymerase chain reaction (PCR) using the following conditions: 12.5 ng DNA; 1X PCR buffer; 0.2 μM of each dNTP; 0.7 μM of each primer (C1236T F: 5^′^-TTGAATGAAGAGTTTCTGATGTTTTC-3^′^, R: 5^′^-CCTGTCCATCAACACTGACC-3^′^; G2677T/A F: 5^′^-TTCTCTAATTTGTTTTGTTTTGCAG-3^′^, R: 5^′^-AAAAGATTGCTTTGAGGAATGG-3^′^; C3435T F: 5^′^-GTGTGCTGGTCCTGAAGTTG-3^′^, R: 5^′^-AGAGAGGCTGCCACATGC-3^′^); 2 mM MgCl_2_ and 0.03 u of Fast Taq polymerase (Roche, Basel, Switzerland) 5-min initial denaturation at 95°C; 35× cycles at 95°C denaturation for 30 s, 58°C annealing for 30 s and 72°C extension for 30 s, and a final extension at 72°C for 10 min. The resulting PCR products were purified by SAP/Exo treatment (GE Healthcare, Little Chalfont, UK), and both strands were sequenced using the Big Dye Terminators v3.0 Sequencing Kit (Life Technologies, Carlsbad, CA, USA) on ABI 3730 (Life Technologies, Carlsbad, CA, USA) and analysed using Seqscape software (Life Technologies, Carlsbad, CA, USA).

### Statistical analysis

Statistical analyses were performed using SPSS 15.0 (SPSS Institute, Chicago, Ill, USA). Values are presented as mean ± standard deviation (SD). Differences in BP_ND_ between groups were assessed using non-parametric Mann–Whitney *U* tests. A general linear model (GLM) with T dose as the continuous measure was used to assess the effects of T dose on BP_ND_. The threshold for significance was set at *p* < 0.05.

## Results

Thirty-two healthy controls (14 females) with a mean age of 47 ± 17 years and seventeen AD patients (5 females) with a mean age of 64 ± 7 years were included in the study.

The prevalence of C1236T, G2677T and C3435T SNPs in both healthy controls and AD patients is listed in Table 1. Of the subjects who were homozygous for the 1236T SNP, 94% were also homozygous for the 2677T SNP. In addition, 73% of the subjects who were homozygous for the 3435T SNP were also homozygous for the 2677T SNP.

**Table 1:** Prevalence of different SNPs in healthy controls and Alzheimer's disease patients

		**HC**	**AD**
C1236T	CC	11 (34%)	3 (18%)
	CT	11 (34%)	8 (47%)
	TT	10 (31%)	6 (35%)
G2677T/A	GG	10 (31%)	3 (18%)
	TG	10 (31%)	8 (47%)
	TT	10 (31%)	6 (35%)
C3435T	CC	8 (25%)	2 (12%)
	CT	15 (47%)	9 (53%)
	TT	9 (28%)	6 (35%)

SNPs, single nucleotide polymorphisms; HC, healthy control group; AD, Alzheimer's disease patient group.

First, healthy controls and AD patients were dichotomized into a group with no T present and a group with one or more T present for C1236T, G2677T and C3435T SNPs separately. For each SNP, (*R*)-[^11^C]verapamil BP_ND_ was compared between these two groups. In healthy controls, no differences in BP_ND_ between these groups were found (Table 2). In AD patients however, significantly higher BP_ND_ values were found in patients with one or more T present in C1236T, G2677T and C3435T SNPs than in those with no T present (Table 2).

**Table 2:** **Global (*R*)-[**^**11**^**C]verapamil BP**_**ND**_**in healthy controls and Alzheimer's disease patients after dichotomization on presence of T**

		**Number**	**HC**	**Number**	**AD**
C1236	no T	11	1.76 ± 0.45	3	1.74 ± 0.33
	T present	21	1.78 ± 0.43	14	2.16 ± 0.29^*^
G2677T/A	no T	11	1.76 ± 0.45	3	1.74 ± 0.33
	T present	21	1.78 ± 0.43	14	2.16 ± 0.29^*^
C3435T	no T	8	1.73 ± 0.49	2	1.56 ± 0.15
	T present	24	1.79 ± 0.42	15	2.15 ± 0.28^*^

HC, healthy control group; AD, Alzheimer's disease patient group; * = *p* < 0.05.

**Figure 1:**
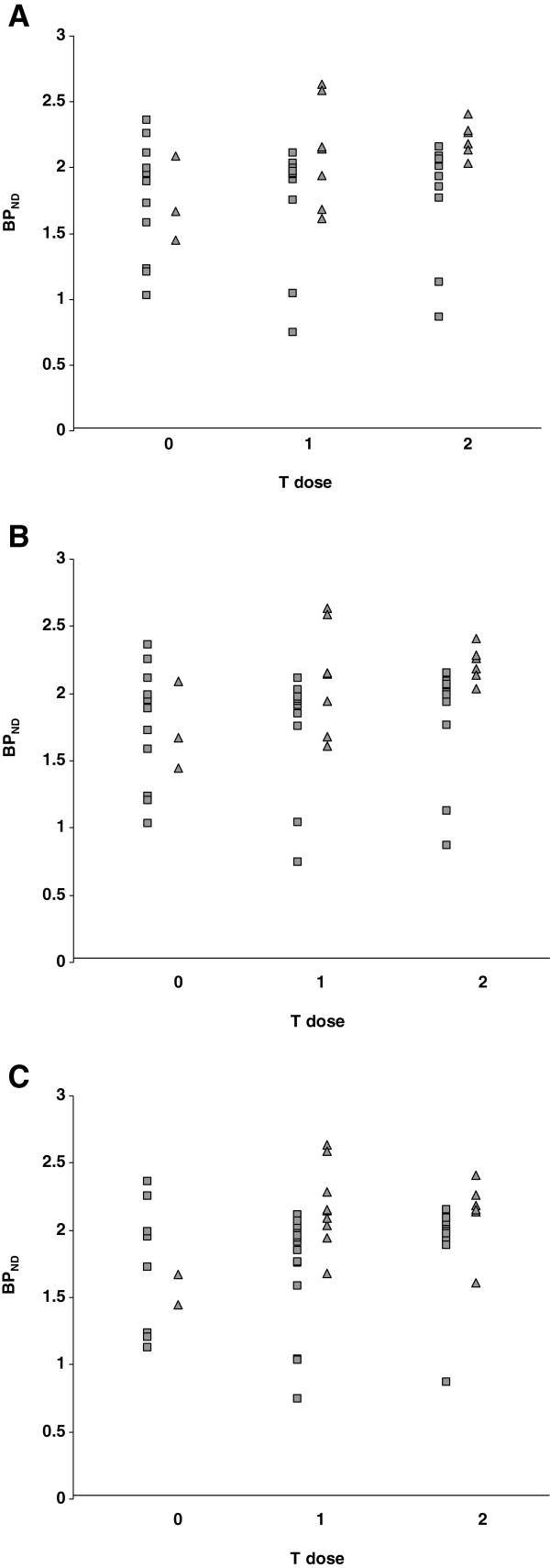
**Binding potential (BP**_**ND**_**) of (*R*)-[**^**11**^**C]verapamil.** Global BP_ND_ in healthy controls (HC, squares) and Alzheimer's disease patients (AD, triangles) as a function of T dose in (**A**) C1236T, (**B**) G2677T and (**C**) C3435T is shown. Mean BP_ND_ values and SD are given. (**A**) C1236T: In HC, BP_ND_ values for 0 T, 1 T and 2 T were 1.76 ± 0.45, 1.77 ± 0.45 and 1.79 ± 0.44, and in AD, the values were 1.74 ± 0.33, 2.11 ± 0.37 and 2.22 ± 0.13, respectively. (**B**) G2677T: In HC, BP_ND_ values for 0 T, 1 T and 2 T were 1.76 ± 0.45, 1.76 ± 0.44 and 1.81 ± 0.44, and in AD, the values were 1.74 ± 0.33, 2.11 ± 0.37 and 2.22 ± 0.13, respectively. (**C**) C3435T: In HC, BP_ND_ values for 0 T, 1 T and 2 T were 1.73 ± 0.49, 1.73 ± 0.44 and 1.88 ± 0.39, and in AD, the values were 1.56 ± 0.15, 2.17 ± 0.30 and 2.13 ± 0.27, respectively.

In addition, the effect of T dose (0 T, 1 T or 2 T present) in C1236T, G2677T and C3435T SNPs was assessed. In healthy controls, no effect of T dose on (*R*)-[^11^C]verapamil BP_ND_ was found (Figure 1). Again, in AD patients, an effect of T dose in C1236T and G2677T SNPs on (*R*)-[^11^C]verapamil BP_ND_ was found (*p* < 0.05), whilst in C3435T SNP, a trend was observed (*p* = 0.14), all with higher BP_ND_ values for higher T dose (Figure 1).

## Discussion

In healthy controls, no effects of SNPs in C1236T, G2677T/A and C3435T on Pgp function at the BBB were found *in vivo*. In contrast, in AD patients, both the presence of T as well as T dose in C1236T, G2677T/A and C3435T were related with increased BP_ND_ of (*R*)-[^11^C]verapamil, suggesting decreased Pgp function at the BBB.

Two previous PET studies, assessing effects of ABCB1 haplotypes on BBB Pgp function in healthy subjects, also did not find differences in the brain uptake of ^11^C]verapamil [16,17]. Before the present study, however, no studies have been performed assessing effects of genetic variations in ABCB1 on *in vivo* BBB Pgp function in AD patients.

The present study showed a selective effect of the C1236T, G2677T and C3435T SNPs in ABCB1 on BBB Pgp function in AD patients. As, using (*R*)-^11^C]verapamil PET, BBB Pgp dysfunction in AD patients has been shown previously [4], the present data suggest that genetic variations in the ABCB1 gene might affect Pgp function or expression at the BBB, only when Pgp function is already compromised. Therefore, in AD patients, genetic variations in ABCB1 could contribute to disease progression as an additional decrease in BBB Pgp function may lead to an increased rate of accumulation of toxic substances, such as amyloid-beta, in the brain. Alternatively, it is also possible that certain genetic variations in ABCB1 have a more primary role in developing neurodegenerative diseases such as AD or Parkinson's disease (PD). For example, in subjects that were exposed to organochlorine insecticides, polymorphisms in ABCB1, associated with decreased ability to clear xenobiotics from the brain, increased the risk of PD [29].

In the present study, the prevalences of different variants in C1236T, G2677T and C3435T SNPs in ABCB1 were comparable between AD patients and healthy controls. A previous pilot study in relatively small groups of demented patients and healthy subjects showed no significant differences in the prevalence of C1236T, G2677T/A and C3435T [30]. Nevertheless, a large population-based study would be needed to assess possible differences in prevalence of genetic variations in ABCB1 between healthy controls and AD patients. Only such a larger study could provide insight into the role of ABCB1 SNPs and haplotypes as a possible risk factor for developing AD. In a subset of such a study, BBB Pgp function should be measured to further evaluate the relationship between ABCB1 SNPs and haplotypes, and BBB Pgp function in both healthy controls and AD patients.

The prevalences of different variants in C1236T, G2677T/A and C3435T in this study were comparable to those reported previously [5,7]. In addition, linkage disequilibrium between the three SNPs, observed in the present study, has also been described before [13-15]. It could very well be that the effects of the different SNPs in ABCB1 that we have found in AD patients are physiologically effectuated through the linkage disequilibrium via the G2677T SNP, which is a non-synonymous variant, thus changing amino acid sequence, while the mutations in C1236T and C3435T are synonymous [5,6].

At present, measuring Pgp function at the BBB *in vivo* can only be performed using (*R*)-[^11^C]verapamil PET with arterial blood sampling. Consequently, group sizes in the present study are limited, and findings need to be replicated in larger trials, preferably using methods without arterial sampling. Another limitation of the present study is the fact that it is not possible to differentiate between decreased Pgp function due to decreased BBB Pgp transporter expression or decreased Pgp transport functionality with intact transporter expression. This can only be addressed when a PET tracer of Pgp expression becomes available.

## Conclusions

Single-nucleotide polymorphisms in C1236T, G2677T and C3435T are related to changes in BBB Pgp function in AD patients, but not in healthy controls. As such, in AD patients, genetic variations in ABCB1 might contribute to the progression of amyloid-beta deposition in the brain.

## Competing interests

The authors declare that they have no competing interests.

## Authors' contributions

DvA acquired the PET data, performed data analysis and wrote the manuscript. ML supervised the PET data analysis. PR performed the DNA isolation and molecular analysis. JvS advised about the use of genetic terminology. RS was involved in quality control of the PET tracer and metabolite analysis and performed quality control of the metabolite data. JE was involved in tracer synthesis and quality control of tracer synthesis processes. PS assisted in recruiting the AD patients. MK was involved in the first ideas for the studies and study design. AL was involved in interpreting data and drafting and improving the manuscript. BvB was the clinical supervisor involved in screening of participants and assisting in the PET data acquisition and helped draft the manuscript. All authors read and approved the final manuscript.
